# Immunotherapeutic implications on targeting the cytokines produced in rhinovirus-induced immunoreactions

**DOI:** 10.3389/falgy.2024.1427762

**Published:** 2024-05-27

**Authors:** Le Sang, Xia Gong, Yunlei Huang, Linling Zhang, Jian Sun

**Affiliations:** ^1^ Department of Medicine, Shaoxing University, Shaoxing City, Zhejiang Province, China; ^2^ Department of Respiratory Medicine, Shaoxing People’s Hospital, Shaoxing City, Zhejiang Province, China

**Keywords:** rhinovirus, rhinovirus-related diseases, immunoreactions, cytokines, therapies for rhinovirus infection

## Abstract

Rhinovirus is a widespread virus associated with several respiratory diseases, especially asthma exacerbation. Currently, there are no accurate therapies for rhinovirus. Encouragingly, it is found that during rhinovirus-induced immunoreactions the levels of certain cytokines in patients' serum will alter. These cytokines may have pivotal pro-inflammatory or anti-inflammatory effects via their specific mechanisms. Thus far, studies have shown that inhibitions of cytokines such as IL-1, IL-4, IL-5, IL-6, IL-13, IL-18, IL-25, and IL-33 may attenuate rhinovirus-induced immunoreactions, thereby relieving rhinovirus infection. Furthermore, such therapeutics for rhinovirus infection can be applied to viruses of other species, with certain practicability.

## Introduction

Rhinovirus (RV), an ubiquitous and widespread respiratory virus, might be crucial in the occurrence and exacerbation of various chronic respiratory diseases, including bronchiectasis, chronic obstructive pulmonary disease, especially asthma exacerbation. Besides, RV is demonstrated as pivotal in wheezing of adults and children. For instance, children who suffer from RV-induced wheezing at an early age are more prone to develop persistent asthma. Also, once asthma is established, RV infections can constantly be a risk factor for acute wheezing illnesses (1, 2). RV is roughly divided into three subtypes (RV-A, B, and C), which have specific receptors, respectively. Generally speaking, it is identified that RV infection activates the Th2 inflammatory response while inhibiting the Th1 inflammatory response (3, 4). Such regularity may indicate that the inhibition of Th2 inflammation can play a role in curing RV infection.

At the same time, it is notable that there are relatively specific changes in pro-inflammatory cytokines and anti-inflammatory cytokines released by immunocytes during RV infection. Therefore, figuring out the relationship between cytokines and RV infection might be helpful for the identification of pathogens. In addition, studies showed that inflammatory response in the host might be attenuated after blocking several pro-inflammatory cytokines (5). Overall, viral infection is considered a self-limited disease. It is reasonable to presume that viral infection can be ameliorated after inhibitions of pro-inflammatory cytokines produced in RV-induced immunoreactions.

This article focuses on the alterations of cytokines during RV-induced immunoreactions and the outcome achieved after inhibitions of these expressed cytokines, trying to search for novel therapies for RV infection.

### RV-related respiratory diseases

RV, a non-enveloped ssRNA virus, belongs to the family *Picornaviridae*, genus *Enterovirus*, and is considered one of the most common independent pathogenic factors of various respiratory diseases.

RV causes respiratory diseases worldwide, and is prevalent all year round, as evidenced by epidemiological investigations (6). The detection rates in certain countries are shown as 33.2%, 50.4%, and 26%, respectively (7–9). In a study, the pathogens in 592 children with community-acquired pneumonia (CAP) confirmed by radiograph were detected. As a result RV was found as an independent agent in 99 cases (29.0%) and as one of the concurrent agents in 73 cases with two or more viruses infected (40.7%) (10). A small sample study showed that approximately 90% of children who had wheezing triggered by RV when they were 3 years old would have asthma when 6 years old (11). It was found that approximately 20% to 40% of bronchiolitis or acute wheezing in infants were caused by RV which as a pathogen was second only to respiratory syncytial virus (RSV), and approximately 60% of acute exacerbation of chronic obstructive pulmonary disease (AECOPD) was also induced by RV (12–15).

Given RV's threat to human health, it is necessary to elucidate the mechanisms of RV infection and find new therapeutics.

## Mechanisms in RV-induced cytokines production

### The viral life cycle of RV

*Picornaviruses* are small, nonenveloped, icosahedral RNA viruses with positive-strand polarity. Even though partial *picornavirus* infections remain asymptomatic, numerous picornaviruses are crucial human and animal pathogens and may lead to diseases which will affect various organs and systems, such as respiratory and gastrointestinal tracts. Thus, the conclusion for the viral life cycle of RV may help understand the bioactivity of RV more deeply (16, 17).

**Figure 1:**
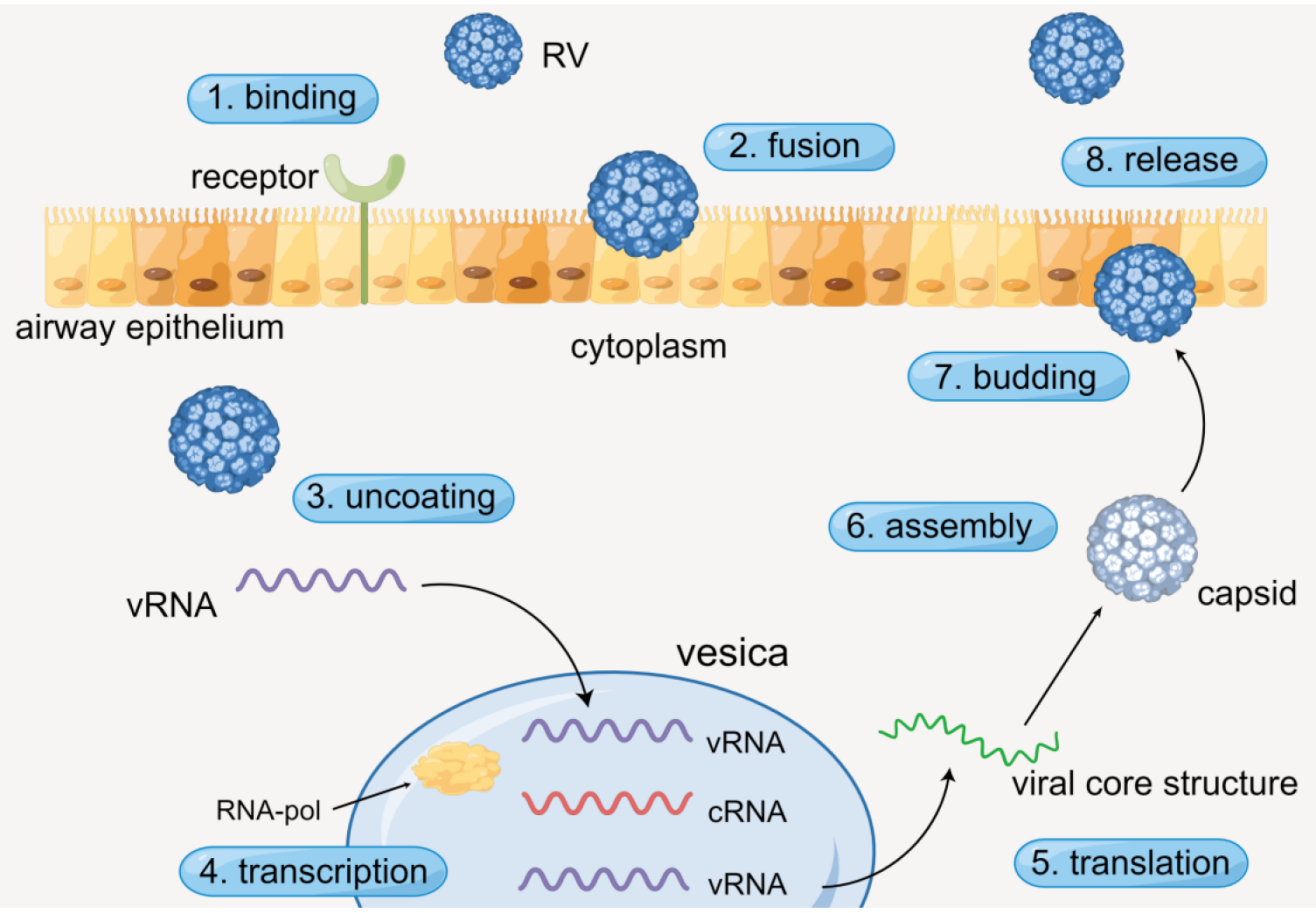
The viral life cycle of RV. By Figdraw.

The process of RV infecting airway epithelium generally contains specific procedures. RV enters the cytoplasm through receptor-mediated endocytosis, and virus RNA (vRNA) is dissociative after viral uncoating. Then the vRNA is transcribed in vesicles with the action of RNA polymerase (RNA-pol). Subsequently, complementary RNA (cRNA) is produced and new vRNA is formed. Ultimately, the new vRNA is translated to form the viral core structure, assembled with the pre-generated capsid, and finally budding and release are completed (Figure 1) (16, 17). Simultaneously, numerous cytokines are released during the process.

### The innate immune mechanism mediated by the toll-like receptors

**Figure 2:**
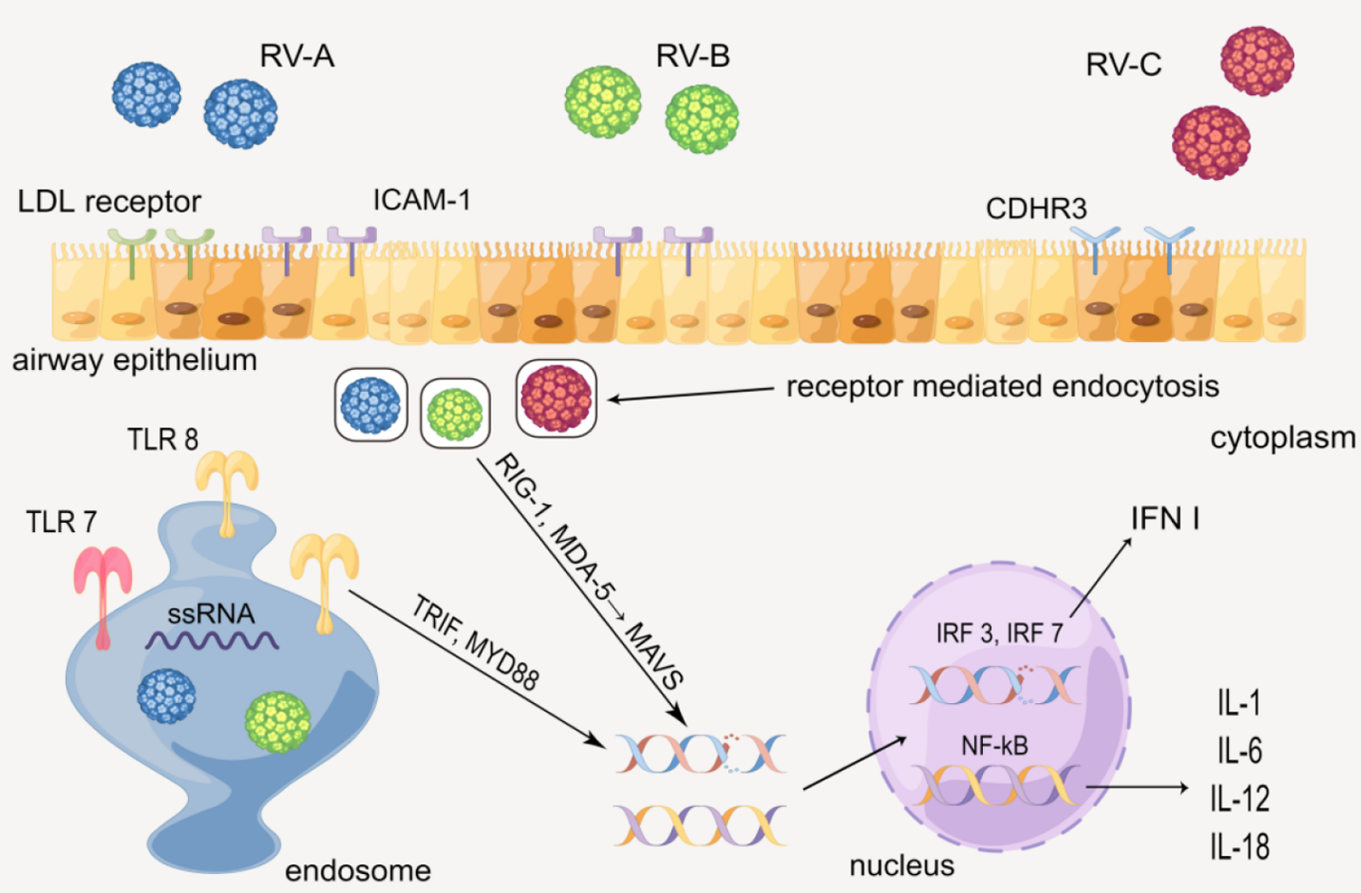
Production of cytokines through the innate immune mechanism mediated by the toll-like receptor. By Figdraw.

When hosts have contact with RV, various cytokines will be produced via the innate immune mechanism mediated by the toll-like receptors. In human airway epithelial cells (HAECs), each of the three types of RV has the distinct receptor: RV-A is taken up by low-density lipoprotein (LDL) receptors, RV-B and much of RV-A are received by intercellular adhesion molecule-1 (ICAM-1), and RV-C is combined by cadherin-related family member 3 (CDHR3) (18, 19). RV enters epithelial cells by receptor-mediated endocytosis, and the ssRNA of RV is recognized by the toll-like receptor (TLR) 7 and 8 in the endosome, which then activates the myeloid differentiation primary response 88 (MYD88) and TIR domain-containing adapter inducing interferon β (TRIF) (1, 19, 20). Besides, RV in the cytoplasm is identified by melanoma differentiation-associated gene 5 (MDA-5) and retinoic acid-inducible gene 1 (RIG-1), activating the mitochondrial anti-viral signaling protein (MAVS) signaling pathway (20–22). MYD88, TRIFF, and MAVS ultimately activate interferon regulatory transfer factor (IRF) 3, 7, and NF-kB, leading to up-regulate the gene expression of cytokines such as IL-1 (IL-1α and IL-1β), IL-6, IL-12, IL-18, and type 1 interferon (INF I) (23–27). The cytokines above subsequently recruit and activate a series of immunocytes such as NK cells, dendritic cells (DC), and T/B cells, causing certain lesions such as airway remodeling (Figure 2) (28–30).

### The acquired immune mechanism

**Figure 3:**
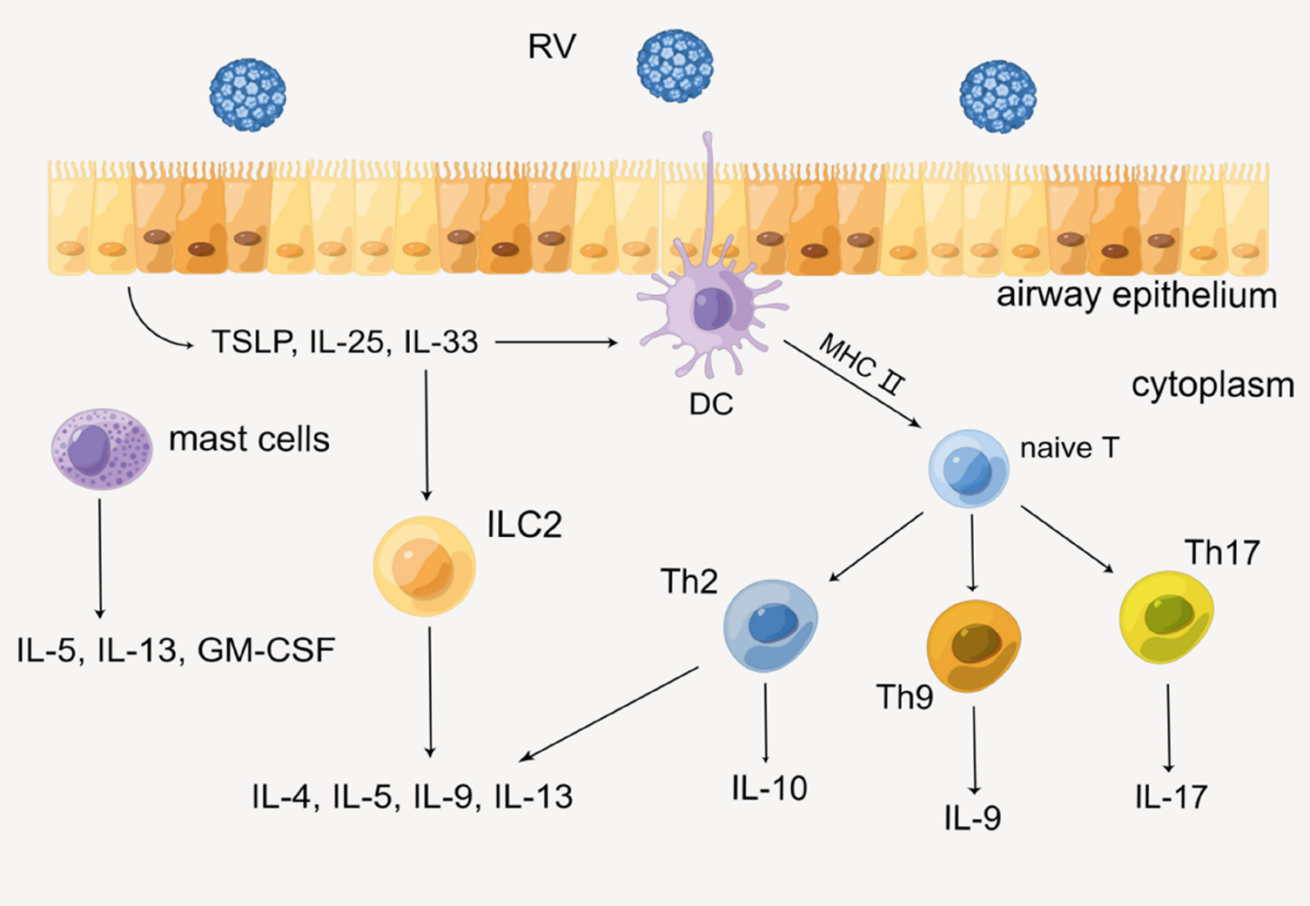
Production of cytokines through the acquired immune mechanism. By Figdraw.

On the other hand, acquired immunity is also indispensable in the secretion of cytokines. Epithelial cells immediately produce several alarmins such as IL-25, IL-33, and TSLP when RV invades HAECs, then recruiting innate lymphoid cell precursors (ILCP) and leading it to differentiate into ILC2 by up-regulating GATA3, retinoic acid receptor-related orphan receptor a (RORa), and T-cell factor 1 (TCF-1), etc. (31, 32). Subsequently, ILC2 produces Th2 cytokines such as IL-4, IL-5, IL-9, and IL-13. In addition, DC is also necessary for acquired immunity. As a professional antigen-presenting cell, DC presents antigen to naive T cells via the MHC class II molecular pathway. With the action of IL-4, naive T cells differentiate into helper T cells (Th) such as Th2, Th9, and Th17, then various cytokines such as IL-9, IL-17, and IFN are produced. In this process, certain immunocytes such as mast cells will also secrete IL-5, IL-13, and granulocyte-macrophage colony-stimulating factor (GM-CSF) (33). Eventually, sequential cytokines lead to the infiltration of a series of immunocytes and inflammatory response, which might impair lung function ultimately (Figure 3) (26, 29, 34).

Taken together, the alterations of cytokines during RV infection might be traceable. Therefore, searching the relationship between alterations of cytokines and viral infection might help identify the pathogens. For instance, RV and influenza virus (IFV) are common viruses causing similar diseases and symptoms. The level of IL-5 in patients' serum during RV infection was found to be higher than that of patients infected with IFV. Detecting the level of IL-5 might be utilized to distinguish between RV and IFV infection (35). In addition, a study showed that the level of RV-induced IL-6 generally peaked at 48 h postinfection. As compared with RSV infection, IL-8 increased more rapidly after RV-C infection. Besides, the levels of RV-induced IL-6 and IL-8 were lower than those induced by RSV. Another recent study also showed that the type of immunoreactions depends on RV serotypes. For example, as compared with RV-16, RV-1B produced higher IFN-β, IFN-λ1/3, CXCL10, IL-6, IL-8, and IL-18 levels (36). Accordingly, partially identifying pathogens by comparing the cytokines' alterations might be feasible, which can direct empirical clinical work to an extent (37).

## Cytokines produced in RV-induced immunoreactions

### Inhibitions of pro-inflammatory cytokines

Therapies for RV infection through anti-interleukin may be practicable. For example, IL-1 is a pivotal inflammatory cytokine leading to neutrophil aggregation, mucus metaplasia, and inflammatory reactions. In the study of Samuel et al, it was found that IL-1α and IL-1β were increased in supernatant and associated with cell death following RV infection (38). It was found that blocking the IL-1 signaling pathway significantly reduced the RV-induced cytokines, consistent with the experimental results of Persson et al. (26). In their research, they found that the main neutrophil chemotactic protein, CXCL1/KC, was less induced at asthma exacerbation in IL-1β knockout mouse models. At the same time, deficiency in IL-1β showed the tendency towards reduced induction of TSLP and IL-33 gene levels. These phenomena may be associated with the amelioration of RV-induced exacerbation of asthma (39–41). Besides, studies showed that dual-specificity phosphatases (DUSP) derived from primary bronchial epithelial cells inhibited IL-1β-mediated inflammatory response, showing the feasibility of therapies for RV infection targeting IL-1. The idea was also confirmed in patients with IFV infection (5, 42, 43). Nowadays, there have been several IL-1β-neutralising biologicals such as anakinra and canakinumab, and their effects in therapies for RV infection still need further clarification.

IL-4 and IL-13 are generally produced in the inflammatory reactions with ILC2 as the core (39). Previous research demonstrated that IL-4 produced by non-T cells was crucial for the aggregation of Th2 cells (33). Another study showed that IL-4 and IL-13 produced remarkable dose-dependent inhibition of RV-induced IFN-β mRNA expression and IFN-β protein release at 8 h after infection, which may partially explain why IL-4/IL-13 promotes the RV infection (44). Meanwhile, a study found that the tight junctions of lung epithelium were destroyed, and immunoreactions against RV were weakened by long-term exposure to IL-13. What's worse, lung function can be damaged (45). Fortunately, targeting IL-4 and IL-13 in therapies for RV infection is highly likely achievable. For instance, blocking IL-13 might alleviate RV-induced diseases in children (46). And the hospitalization rate of asthmatics was reduced after administrating the IL-4 antagonist (dupilumab) and IL-13 antagonist (lebrikizumab or tralokinumab). It is reasonable to assume that L-4 and IL-13 blockade decreases the Th2 response so that they relieve RV-related diseases, which may be verified in the research of David et al. They found that anti-IL-4, anti-IL-13, and anti-IL-4/13 reduced airway inflammation. Besides, it might be more significant to administrate with anti-IL-4/13 concurrently. The mice treated with combined anti-IL-4/13 were more manifestly protected from arteriolar hypertrophy and fibrosis, whereas mice treated with anti-IL-4 less suffered from fibrosis and still had evidence of arteriolar hypertrophy (47). Yet, in the meanwhile, it was shown that IL-4 and 13 antagonists may increase the risk of adverse events as compared with patients utilizing IL-5 and IgE antagonists, needing further study (48).

IL-5 is mainly secreted by Th2 immunocytes, and related to severe asthma. There is a higher level of IL-5 in asthmatics’ serum after RV infection (23, 49). Currently, several monoclonal antibodies against IL-5 show positive effects. For instance, mepolizumab and reslizumab show the ability to inhibit the binding of IL-5 and IL-5R, while benralizumab directly inhibits IL-5Rα (50). The medicine above reduces the eosinophils in patients’ peripheral blood, which might relieve RV-induced asthma. In November 2021, mepolizumab was licensed by China's Drug Administration (NMPA) for utilization to treat adult eosinophilic granulomatous polyangiitis. Similarly, it is believable that treatments for RV infection via anti-interleukin might also have feasibility. In addition, a study showed that after asthmatics utilized IL-5 antagonists, there were more IFN-α secreted by plasmacytoid dendritic cells (pDC) in patients' serum, inspiring that anti-IL-5 can be effective for curing RV-related diseases such as asthma (51).

Neutrophils-mediated epithelial airway damage and airway hyper-responsiveness (AHR), induced by RV, might be triggered by IL-6 and IL-8 (37). IL-6 is produced by several immunocytes such as macrophages and DC. And it has two types of receptors (IL-6R): membrane-bound IL-6R and soluble IL-6R, cooperating with TGF-β to boost the differentiation of Th17 cells (52–54). Regarding IL-6 antagonists, Tocilizumab has been routinely utilized to treat rheumatic immune diseases, showing great effectiveness. Besides, other available IL-6 inhibitors such as sirukumab, olokizumab, and siltuximab are limited to utilization due to their high cost, invasive delivery techniques, and high immunogenicity. Therefore, novel IL-6 inhibitors are required. Generally, there are three types of IL-6 inhibitors, namely IL-6 production inhibitors, IL-6 expression inhibitors, and IL-6 signaling pathway inhibitors. Encouragingly, several novel IL-6 antagonists have been found. For example, phenyl ring might reduce the level of LPS-induced IL-6, steroids from marine organisms might inhibit the IL-6 mRNA expression, epoxyresibufogenin-3-formates might have the pharmacological effect to antagonize IL-6R, and thiophene derivatives might be IL-6-induced STAT3 inhibitors (54). However, even though new IL-6 antagonists emerge endlessly, their clinical application in RV-related diseases lacks enough research.

IL-11, belongs to the IL-6 family, with pro-inflammatory and anti-inflammatory effects concurrently (55, 56). During RV infection, IL-11 is also released (23, 24). IL-11 and α-IL-11R form a compound with glycoprotein 130, activating the downstream. signal pathway, and finally exerting biological effects (57, 58). It is believed that IL-11 is redundant in adults, and may lead to idiopathic pulmonary fibrosis and asthma (59–62). In addition, treatments for RV infection targeting IL-11 show possibilities due to the combination between IL-11 and its receptor is likely to be blocked (61, 63). For instance, a study showed that recombinant mouse IL-11Rα Fc chimeric protein, an IL-11 antagonist, inhibited Th17 cell-mediated neuroinflammatory response (64). However, there is insufficient research on the relationship between IL-11 and RV infection.

IL-15, containing a series of cytokines, is produced by certain antigen-presenting cells such as DC, monocytes, and macrophages during RV infection. The productions of natural kill cells, CD8+ T cells, and IFN-γ are stimulated after IL-15 binds to its receptor, thus attenuating viral infection (65, 66). A study showed that IL-15 isoform suppressed the effect of IL-15 in several inflammatory diseases (67). Yet, the compound of IL-15 and its receptor (sIL-15Rα) had no positive impact on the lung function of asthmatic mice (68).

Previous research showed that after RV infection, the level of IL-17 secreted by various immunocytes including mast cells and macrophages would increase, leading to neutrophil recruitment and AHR, even damaging lung function (69, 70). Currently, the monoclonal antibodies against IL-17 such as secukinumab, lxekizumab, and brodalumab are utilized to cure diseases such as psoriasis, among which secukinumab has been approved by the FDA. Additionally, neutralizing IL-13 and IL-17 simultaneously may positively affect asthmatics from eosinophilia, mucus hyperplasia, and airway hyperreactivity and abolish the neutrophilic inflammation, indicating the potential of blocking IL-13 and IL-17 for therapies for RV infection (47).

IL-18, as an IFN-γ inducible factor related to RV, is produced by inﬂammasome, and might enhance epithelial cell differentiation (25, 71). The Th2 inflammatory and allergic reactions might be promoted after IL-18 combines with its receptor (IL-18R) (72, 73). And it was found that IL-18 increased in severe asthmatic sputum and serum (74). In addition, the IL-18 axis also exists in severe asthma as proved by machine learning. Also, an endogenous antagonist against IL-18, IL-18 binding protein (IL-18BP), might neutralize IL-18 and IL-18R to inhibit the biological effects (75). Accordingly, blocking IL-18 to ameliorate RV infection can be practicable.

Thus far, research shows that HAECs from asthmatics release IL-25 during RV infection, enhancing Th2 inflammation and closely related to severe asthma (76, 77). Regarding IL-25 antagonists, LNR125 was confirmed. to relieve viral infection by up-regulating type I&III interferon genes and down-regulating type II inflammatory genes such as CCL26, IL1RL1, and IL-25 receptor, verifying that the therapies for RV infection in the basis of anti-IL-25 might be feasible (78).

Several studies show that IL-33 might have the capability of increasing the production of Th2 cytokines such as IL-4, IL-5, and IL-13, and promote RV-induced inflammation (79). For example, IL-33 boosts the release of IL-5 and IL-13 and the virus-specific Th2 inflammation via the IL-33/ST2 (IL-33R) signaling axis in allergic asthmatics (80, 81). Also, IL-33 was detected in the supernatant of HAECs from RV-infected patients, while the phenomenon was entirely repressed after blocking IL-33, indicating that anti-IL-33 might be utilized in treatments for RV infection (39). The results are consistent with the previous conclusion concerning anti-IL-33 (itepekimab) (82). Besides, 25 (OH)-Vitamin D3 [25 (OH)-VitD3] has also been proven to have the effect of antagonizing ST2 (83, 84). In addition, in asthmatic children with RV infection, soluble ST2 which may neutralize IL-33 is significantly reduced by low serum levels of 25 (OH)-VitD3. The low level of 25 (OH)-VitD3 might reduce the production of interferon β (IFN-β) (85). Thus, 25 (OH)-VitD3 can be applied to therapies for RV infection in the future.

Currently, studies show that RV infection might activate peripheral transforming growth factor β (TGF-β), which might subsequently recruit neutrophils and monocytes, and induce Th17 cells and regulatory T cells (Tregs) through downstream. forkhead box P3+ (FOXP3) and retinoic acid-related orphan receptor γ (RORγ), ultimately causing airway remodeling and inflammation. After RV infection, the combination of TGF-β and its receptor on the cell surfaces increases, promoting viral replication and the severity of asthma (52). Besides, it was found that the inflammation was alleviated after the TGF-β pathway was antagonized (86). Therefore, it might be feasible to block TGF-β for therapies for RV infection.

The IFN-β/programmed death ligand-1 (PD-L1) axis also affects asthmatic children with RV infection. A short cohort study showed that in the control group, the IFN released by PBMC was related to the production of PD-L1. Yet, the study also showed that higher IFN-β levels were associated with the lower level of PD-L1 and better lung function (87). To summarize, PD-L1 might be a pro-inflammatory cytokine in RV-related asthmatics. Currently, PD-L1 has been widely utilized in anti-tumor immunotherapies, but whether it can be utilized in therapies for RV infection requires further research (Table 1).

**Table 1:** Pro-inflammatory cytokines.

Cytokines	Changes	Representative antagonists	Effects or potentials	References
IL-1	↑	DUSP, anakinra, canakinumab	√	(5, 26, 38, 42, 43)
IL-4	↑	Dupilumab	√	(44, 45)
IL-5	↑	Mepolizumab, reslizumab, benralizumab	√	(23, 49, 51)
IL-6	↑	Sirukumab, olokizumab, siltuximab, phenyl ring, epoxyresibufogenin-3-formates	√	(37, 52, 53)
IL-11	↑	Recombinant mouse IL-11Rα Fc chimeric protein	/	(55, 56, 64)
IL-12	↑	Ustekinumab	/	(23–27)
IL-13	↑	Lebrikizumab, tralokinumab	√	(46, 48)
IL-15	↑	IL-15 isoform	/	(65, 66, 68)
IL-17	↑	Secukinumab, lxekizumab, brodalumab	/	(47, 69, 70)
IL-18	↑	IL-18BP	√	(25, 71, 75)
IL-25	↑	LNR125	√	(76–78)
IL-33	↑	Itepekimab	√	(39, 79)
TGF-β	↑	1D11	√	(52, 86)
PD-L1	↓	Pembrolizumab, nivolumab	/	(87)

IL, interleukin; DUSP, dual-specificity phosphatases; IL-18BP, IL-18 bind protein; TGF-β, transforming growth factor β; 1D11, anti-TGF-β; PD-L1, programmed death ligand-1.

## Utilizations of anti-inflammatory cytokines

Several anti-inflammatory cytokines are pivotal in immunoreactions against RV. It is quite necessary to figure out their alterations and effects due to their potentials for curing RV infection.

The level of GM-CSF is affected by diversified factors. For instance, GM-CSF is produced by epithelial cells, fibroblasts, and mast cells stimulated by RV (88–90). And the production can be inhibited by certain cytokines such as IFN-γ, IL-1β, IL-4, and IL-10 (91–93). GM-CSF activates the downstream, Janus kinase (JAK) 2, STAT5, and the phosphatidylinositol-3-kinase (PI3K) pathway after binding to its receptor, then leading to the recruitment and activation of several inflammatory cells such as monocyte macrophages and DC (94–96). A study showed that inhaled sargramostim (yeast-derived recombinant human GM-CSF) might significantly improve oxygenation in hospitalized COVID-19 patients with hypoxic respiratory failure, and subcutaneous injection of sargramostim might shorten mechanical ventilation of patients with severe sepsis (97, 98). By analogy, GM-CSF probably has the potential for treatments for RV infection.

IFN can also be produced by pDC in RV-induced immunoreactions (99). It appears that IFN-α and IFN-β couple to the same receptor, which is different from the receptor for IFN-λ. Type I IFN (IFN-α and IFN-β) receptors (IFNAR), are coupled to the JAK1 and the Tyrosine kinase 2 (TYK2). After Type I IFN binds to IFNAR, JAK1 and TYK2 are activated, which then phosphorate signal translator and activator of translation (STAT) 1 and 2. The compound of STAT homodimers and interferon-stimulated gene factor (ISGF) 3 forms, eventually inducing ISG transcription in the nucleus (100–102). The process above has the effects against viral infection. Type I IFN also recruits immunocytes such as NK cells and DC, exerting immunifaction (103, 104). In addition, Type I IFN signaling induces the production of the 2′-5′-oligoadenylate synthetase (OAS) family, which includes OAS1, OAS2, OAS3 and OAS-like (OASL) proteins. It was identified that OASL is manifestly induced upon viral infection through the involvement of the RNA sensor, to promote the anti-viral type I IFN response (105). In several studies, deficiency of various IFN has been verified utilizing cultured human bronchial epithelial cells (HBECs) from asthmatics after RV infection *in vitro*, which leads to the deficiency of IFN-β and IFN-λ in primary bronchial epithelial cells (PBECs) and deficiency of IFNs (γ, α, β and λ) and IL-15 in bronchoalveolar lavage (BAL) cells (23). This result is accorded with that of Lin et al, showing the feasibility of utilizing IFN for alleviating RV infection (106).

IL-10 was identified over two decades ago and the most studied suppressive molecule of the immune system so far. IL-10, found to be secreted by Th2 cells, plays a critical role in anti-inflammatory and autoimmune pathologies by limiting immunoreactions to pathogens (107). Thus far, several studies show that IL-10 is likely to be produced by various cells (108, 109). As an essential anti-inflammatory cytokine family released in RV-induced immunifaction, it includes IL-9, IL-20, IL-22, IL-24, IL-26, IL-28A, IL-28B, and IL-29 (110, 111). The IL-10/IL-10R axis exerts effects through the downstream. mechanism similar to that of Type I IFN (112–114). The IL-10/IL-10R axis triggers a series of signaling cascades mediated by the JAK signal transducer and activator of the transcription (STAT) pathway, especially by STAT3. signaling through the IL-10/IL-10R axis regulates several steps of the immune response, from decreasing cytokine gene expression to inhibiting the antigen-presenting ability of monocytes via down-regulating the MHC class II molecules (115). Besides, IL-10 has been found to have the capability of preventing apoptosis by enhancing the PI3K/Protein Kinase B (AKT) cascade and promoting the expression of anti-apoptotic factors as Bcl-2 and Bcl-xl, while weakening that of caspase-3 (109). In the meantime, IL-10 attenuates the toxicity of pathogens, and reduces the cytokines released by macrophages and DC, thus producing anti-inflammatory effects (116–118). Generally, IL-10 is considered crucial in anti-inflammation. Its clinical application in treating RV infection is worthy of further study.

It was found that under the action of several pro-inflammatory cytokines, HAECs will secrete several IL-1 receptor antagonists (IL-1RA) such as secreted IL-1RA (sIL-1RA) and intracellular IL-1Ra (icIL-1Ra). Among them, Levine et al. proposed that icIL-1Ra type I release from HAEC may modulate IL-1 bioactivity in the airway microenvironment, weakening inflammation subsequently (119). The phenomenon indicates whether there are more endogenous antagonists of pro-inflammatory cytokines in the host, requiring more exploration (Table 2).

**Table 2:** Anti-inflammatory cytokines.

Cytokines	Changes	Effects or potentials	References
GM-CSF	↑	√	(88–90, 97, 98)
IFN I	↓	√	(103, 104, 106)
IL-10	↑	√	(116–118)
IL-1RA	↑	√	(119)

GM-CSF, granular macrophage colony-stimulating factor; IL, interleukin; IL-1RA, IL-1 receptor antagonists; IFN I, interferon type 1.

## Conclusion

The threat from respiratory viruses is nonnegligible due to their capacity to cause various diseases. RV, as a common respiratory virus, is closely related to chronic respiratory diseases, especially asthma exacerbation. Currently, several experiments show that during RV-induced immunoreactions the levels of several cytokines in the hosts' serum will alter, indicating whether it is achievable to identify pathogens according to such alterations. In general, viral infection is known as a self-limited disease, which may drive us to assume viral infection can be ameliorated after the virus-induced immunoreactions are inhibited. Fortunately, researchers find that inflammatory response is inhibited after blocking several pro-inflammatory cytokines such as IL-1, IL-4, IL-5, IL-6, IL-25, and IL-33, showing the feasibility of therapies for RV infection targeting RV-induced cytokines. Several endogenous cytokines such as IL-10, Type I IFN, and IL-1RA might inhibit RV-induced inflammatory response, with practicability in treatments for RV infection.

## Future perspective

RV is crucial in respiratory diseases, and also positively associated with asthma severity. However, therapeutics targeting RV are deficient, and it is difficult to identify the similar respiratory pathogens. Searching for novel and accurate methods of pathogenic diagnosis is necessary, which may guide empirical therapies in clinical work. In numerous experiments, researchers have found that the levels of cytokines in patients' serum after RV infection will change. What's more, these cytokines may be antagonized or directly utilized to attenuate RV infection. It is worthy of summarizing such cytokines' changes and searching for therapies for RV infection.

Currently, research shows that several antagonists of pro-inflammatory cytokines have manifest potentials for anti-inflammation. For example, mepolizumab, the IL-5 antagonist, has been widely utilized to treat eosinophilic granuloma. In the future, it might be applied to cure RV-related diseases. Besides, additional statistics concerning RV-induced cytokine alterations are required to further clarify the relationship between cytokines and RV infection.

In general, the pro-inflammatory cytokines mentioned in the article boost inflammation during RV infection. Yet, the opposite conclusion was reached after administrating IL-1β inhibitors such as NLRP3 KO in immature mice, that RV-induced type II immunoreactions were aggravated (120). It indicated that whether several cytokines such as IL-1 were indispensable in establishing immunity in immature individuals, is worthy of more research.

Even though RV is prevalent widely, its virulence is relatively weak. While RV induces relatively specific alterations of cytokines, different viruses may induce similar immune reactions to an extent. Therefore, it is reasonable to propose that therapies for RV infection targeting cytokines can also be applied to other more threatening viruses. For instance, tocilizumab has been approved to treat COVID-19 by the FDA and NMPA, similarly verifying the practicability of such therapies for RV infection.
